# Sex and age effects on binge eating spectrum disorders in young adults: a path analysis

**DOI:** 10.3389/fpsyg.2026.1870692

**Published:** 2026-09-22

**Authors:** Geovanny Genaro Reivan-Ortiz, María Quezada Gómez, Roser Granero

**Affiliations:** 1 Catholic University of Cuenca, Cuenca, Azuay, Ecuador; 2 Ministerio de Salud Publica de Ecuador, Quito, Pichincha, Ecuador; 3 Department of Psychobiology and Methodology, Universitat Autònoma de Barcelona, Barcelona, Spain

**Keywords:** age, binge eating disorder, binge eating spectrum, bulimia nervosa, night eating syndrome, path analysis, sex

## Abstract

**Background and aims:**

Binge spectrum eating disorders (BSED) are heterogeneous conditions characterized by recurrent binge eating and associated features. While demographic factors are known to influence eating disorder expression, the specific contributions of sex and age remain unclear. This study examined the impact of sex and age on the clinical presentation of BSED in young adults and explored the underlying pathways using path analysis.

**Method:**

The sample comprised 187 young adults (119 women, 68 men; 20–45 years) diagnosed with bulimia nervosa (BN, *N* = 41), binge eating disorder (BED, *N* = 35), night eating syndrome (NES, *N* = 30), or OSFED with binge episodes (*N* = 81). Measures included eating disorder symptomatology, emotion regulation, impulsivity, drive-to-eat behaviors (food cues, food craving and hunger), and psychological state (anxiety-depression, obsessive-compulsive and perceived stress).

**Results:**

Sex and age differences were observed only for hunger and food cue responsiveness, with women reporting higher hunger and younger men higher cue responsiveness; no differences emerged for emotion regulation, impulsivity, or psychopathology. Path analysis revealed direct and indirect effects of sex and age on drive-to-eat behaviors and psychopathology, with emotional regulation mediating pathways in women and impulsivity mediating pathways in older adults, linking appetite-related responses to higher psychological distress and eating disorder severity.

**Conclusion:**

Sex and age shape the clinical expression of BSED in young adulthood. Understanding how these demographic factors interact with psychological and behavioral characteristics may enhance assessment and support the development of tailored interventions across the binge eating spectrum.

## Highlights

- Sex and age were associated with distinct drive-to-eat patterns in the binge spectrum eating disorder.
- Women showed greater hunger, while younger men showed higher food cue responsiveness.
- Emotion regulation emerged as a key pathway linking female sex to clinical outcomes.
- Impulsivity was a central pathway linking older age to food-related and clinical outcomes.
- Distinct pathways may inform more tailored assessment of binge spectrum eating disorder in young adults.

## 1 Introduction

Binge-spectrum eating disorders (BSED) comprise a group of eating-related conditions characterized by a perceived loss of control over food intake which can sometimes be followed by inappropriate weight-control behaviors (such as self-induced vomiting or excessive, unhealthy exercise) (American Psychiatric Association, 2013). This spectrum includes diagnoses such as bulimia nervosa (BN), binge eating disorder (BED), night eating syndrome (NES), and other specified feeding and eating disorders (OSFED) involving binge episodes. These conditions share the core feature of binge eating and evidence similarities in their clinical manifestations, associated psychological characteristics, and behavioral patterns (Giel et al., 2022). Moreover, BSED are increasingly conceptualized by clinicians and physicians within an overeating continuum, in which nonpathological overeating behaviors lie at one end and BSED represents the pathological and more extreme end (Davis, 2013). Progression along this continuum thus reflects increasing severity and compulsivity in overeating behaviors (Arexis et al., 2023).

Accumulating evidence indicates that disorders within the BSED are associated with a broad range of adverse health outcomes (Treasure et al., 2022; van Hoeken and Hoek, 2020). From a physical health perspective, these conditions are linked to increased risk of being overweight and obesity (Gardizy et al., 2024), as well as metabolic complications including Type 2 diabetes (Raevuori et al., 2015) and broader metabolic dysregulation, such as components of the metabolic syndrome and elevated cardiovascular risk (Solmi et al., 2021; Yoon et al., 2019). Gastrointestinal disturbances are also commonly reported, likely reflecting both behavioral and physiological consequences of disordered eating (Hetterich et al., 2019). From a psychological standpoint, BSED show strong comorbidity with psychiatric conditions (Udo and Grilo, 2019) and an increased risk of suicidality (Brown et al., 2018). Overall, these conditions are consistently associated with reduced health-related quality of life across multiple domains, as well as with higher health care utilization and costs (Ling et al., 2017).

A growing body of research suggests that binge eating behaviors arise from the interaction of multiple psychological and motivational mechanisms rather than from a single causal factor (Dufour et al., 2026). Within this framework, two relatively stable individual characteristics have been consistently identified as central vulnerability mechanisms: difficulties in emotion regulation and high impulsivity. Difficulties in emotion regulation have been conceptualized as a multidimensional construct encompassing several components, including nonacceptance of emotional responses, difficulties in engaging in goal-directed behavior, impulse control difficulties, limited emotional awareness, restricted access to effective regulation strategies, and a lack of emotional clarity (Gratz and Roemer, 2004). Moreover, emotion regulation is widely recognized as a transdiagnostic factor that contributes to both the development and maintenance of eating disorders (Arexis et al., 2023; Mendia et al., 2026; McClure et al., 2022; Prefit et al., 2019; Russell et al., 2017). Individuals who struggle to regulate negative moods may rely on maladaptive coping strategies, including overeating or binge eating, as a way to alleviate or temporarily escape distressing emotional states (Braden et al., 2018; Dingemans et al., 2017; Lampe et al., 2026; Svaldi et al., 2019; Walenda et al., 2021). Within the BSED, it has been observed that higher emotion regulation difficulties are related to more frequent compensatory behaviors and greater restraint, shape concern and global eating pathology (Racine et al., 2015; Racine and Horvath, 2018; Riddle et al., 2023).

Impulsivity has been identified as a significant transdiagnostic construct for the understanding and diagnosis of a variety of psychological illnesses (Hamilton et al., 2015; Maerean et al., 2025; Petrovskiy et al., 2026). Classical models define impulsivity as the predisposition toward rapid and poorly controlled behavioral responses, often occurring without adequate consideration of potential consequences (Swann et al., 2002). More recent models of impulsivity have concluded that reward sensitivity and disinhibition are the most influential aspects in explaining addictive-like behaviors (Gullo et al., 2014), since those observed in patients within the BSED. It has been observed that these conditions exhibit elevated impulsivity associated with diminished inhibitory control and greater susceptibility to disinhibited eating patterns (food-related impulsivity) (Giel et al., 2017; Saruco and Pleger, 2021), and this pattern is closely tied to excessive consumption and binge-eating behaviors (Boswell and Grilo, 2021; Carr et al., 2021; İnce et al., 2021; Leehr et al., 2018). Research addressing the neurobiological aspects of binge eating behaviors have also highlighted increased attentional bias for food cues, alterations in reward pathways, and deficits in executive functioning such as poor inhibitory control over food-related impulses (Celeghin et al., 2023; Kessler et al., 2016; Smith et al., 2020; Waltmann et al., 2021).

Eating behavior patterns for individuals with BSED are also associated with proximal motivational processes related to the drive-to-eat, such as hunger, food craving, and responsiveness to food cues (Boswell and Kober, 2016; Stojek and MacKillop, 2017; Wonderlich et al., 2017). Hunger reflects the physiological and subjective experience of energy need, whereas food craving refers to an intense and often specific desire for palatable foods. Food cue responsiveness, in turn, captures the motivational and attentional processes triggered by environmental stimuli related to food availability, such as visual or olfactory cues. Studies have concluded that food cues represent conditioned stimuli that trigger a conditioned response (food craving) and that these associations are stronger in individuals with binge eating behaviors as compared to healthy controls (Meule et al., 2018). Also, it has been observed that psychological traits such as impulsivity and emotion regulation difficulties may shape the intensity and expression of these appetite-related responses. For example, individuals with impaired emotion regulation may experience stronger craving states or may use food consumption as a strategy to cope with negative emotional experiences (Evers et al., 2018; Leehr et al., 2015). Similarly, individuals with higher impulsivity may exhibit greater sensitivity to rewarding food stimuli and reduced ability to inhibit cue-driven eating behaviors (Schag et al., 2013a; Schag et al., 2013b). In this sense, emotion regulation difficulties and impulsivity can be conceptualized as upstream vulnerability mechanisms that increase susceptibility to drive-to-eat processes, which in turn may contribute to the emergence or exacerbation of binge eating symptoms. Binge eating patterns have been associated to attentional bias toward food stimuli at multiple stages of processing, evidenced both in the rapid, automatic orientation of attention and in the more deliberate difficulty disengaging from such cues (Stojek et al., 2018). Individuals with these eating behaviors orient more quickly toward food-related stimuli and maintain their focus on highly palatable foods for longer periods, and this can be explained by heightened reward sensitivity alongside reduced inhibitory control when responding to food cues (consistent with an approach–approach motivational dynamic) (Leehr et al., 2016). Impairments in inhibitory control, together with elevated impulsivity, may hinder these individuals’ ability to effectively downregulate the processing of salient food-related cues.

Beyond these psychological mechanisms, demographic characteristics may further influence phenotypes within BSED. First, sex differences are well documented, with women generally showing higher prevalence rates (Burnette et al., 2022) and greater levels of emotional distress and eating-related concerns (Shingleton et al., 2015) than men. Some studies suggest that women may be particularly vulnerable to binge eating through affective pathways, including heightened emotional reactivity and greater difficulties in regulating negative emotions (Anversa et al., 2021). In contrast, men may show relatively stronger patterns of cue-driven eating and externally triggered intake, potentially reflecting differences in decision-making and responsiveness to food-related stimuli (Amicis et al., 2023). However, a large proportion of men affected by BSED do not access or receive psychological treatment for their symptoms, and existing research in this area continues to include a markedly low representation of male participants (Coffino et al., 2019; Richardson and Paslakis, 2021). Studies have also observed lower inhibitory control for women with BSED as compared to men (Mallorquí-Bagué et al., 2023). Likewise, systematic reviews have observed that men and women within BSED enrolled in treatment are much more similar than different (Sonnenblick et al., 2026), and similar treatment outcomes have been observed comparing both sexes (Grabauskaitė et al., 2017). Therefore, research examining sex differences in the mechanisms underlying binge eating across the broader spectrum of binge-related disorders remains limited.

Young age is also an important factor in the onset and progression of BSED (Bertoli et al., 2016; Gardizy et al., 2024). The mechanisms involved in these clinical conditions are highly vulnerable to the changes that occur at the end of adolescence and beginning of adulthood. This is a developmental stage characterized by ongoing maturation of self-regulatory abilities (such as inhibitory control, cognitive flexibility, and planning; Westermann et al., 2025), as well as impulse control and emotional management (Félix et al., 2025; Zhou et al., 2025). This period is also marked by an imbalance between relatively earlier maturation of subcortical reward systems and the still-developing prefrontal control regions, which may increase vulnerability to dysregulated eating behaviors (Larsen and Luna, 2018). While impulsivity tends to decline gradually with age, emotion regulation capacities often improve over time as individuals develop more adaptive coping strategies. In addition, heightened reward sensitivity during this stage may amplify responsiveness to internal states such as hunger or craving, as well as to external food-related cues. Moreover, this life stage often involves major transitions (e.g., increased autonomy, changes in living environment, and academic or occupational stress), which may further disrupt eating patterns and self-regulation (Arnett, 2015). In addition, food insecurity (identified as a significant risk factor for binge eating and related behaviors, potentially through cycles of restriction and overeating as well as increased psychological stress), is more prevalent during this transitional period (Bidopia et al., 2023; Hazzard et al., 2020). This period also coincides with increased incidence of mood and anxiety disorders (Solmi et al., 2022), which may interact with emotion regulation difficulties and contribute to binge eating behaviors. Nevertheless, empirical evidence examining the combined influence of age, psychological traits, and appetitive processes in binge eating spectrum disorders remains scarce.

In summary, these considerations suggest a multilevel framework in which demographic characteristics (sex and age) may influence relatively stable psychological traits (emotion regulation and impulsivity), which in turn shape drive-to-eat processes (hunger, food craving, and responsiveness to food cues), ultimately contributing to the severity of eating disorder symptoms and associated psychological distress (see Figure 1). Analytical approaches capable of examining these relationships simultaneously may therefore provide a more comprehensive understanding of the mechanisms underlying the clinical presentation of BSED. Structural equation modeling and path analysis offer suitable tools for examining such multivariate relationships, as they allow the estimation of both direct and indirect pathways among variables. Applying this approach to the study of BSED may help clarify how demographic factors interact with psychological traits and appetitive processes to influence psychopathology and eating disorder severity. Identifying these pathways may also have clinical relevance by helping clinicians recognize sex- and age-related patterns in the clinical presentation of BSED and by informing more individualized assessment and intervention strategies.

**Figure 1:**
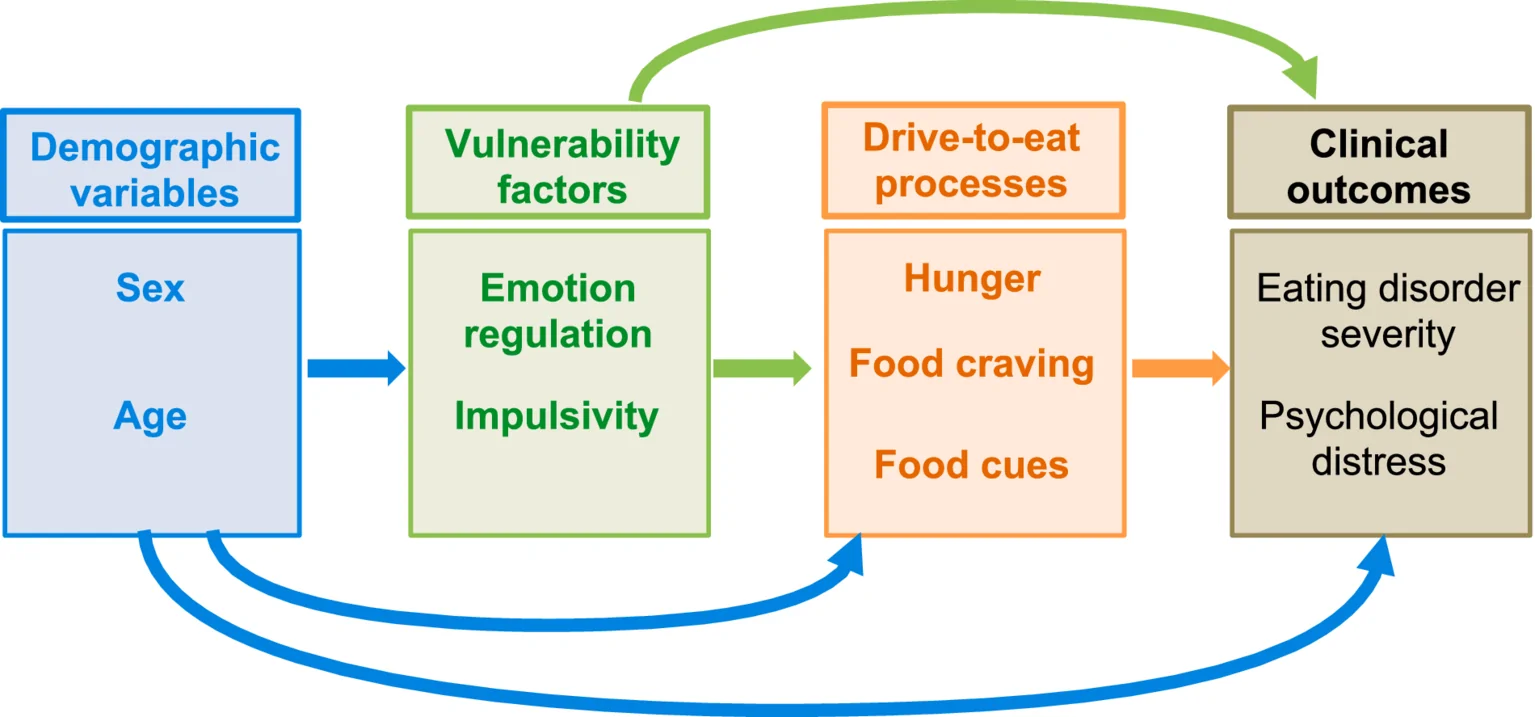
Conceptual framework illustrating the hypothesized relationships among demographic variables, psychological vulnerability factors, drive-to-eat processes, and clinical outcomes.

Therefore, the present study aimed to examine the role of sex and age in shaping the clinical presentation of BSED among young adults diagnosed with BN, BED, NES, or OSFED with binge episodes. In addition, the study sought to explore the underlying relationships between sex and age with emotion regulation, impulsivity, drive-to-eat behaviors (hunger, food craving, and food cue responsiveness), and psychological distress using path analysis.

Based on the theoretical framework described above, several hypotheses were proposed: (H1) sex and age would be associated with differences in drive-to-eat processes, with women expected to report higher levels of hunger and younger individuals showing greater responsiveness to food-related cues, based on previous evidence of sex differences in affective and eating-related processes and developmental changes in reward sensitivity and self-regulation (Amicis et al., 2023; Anversa et al., 2021; Larsen and Luna, 2018; Westermann et al., 2025); (H2) emotion regulation difficulties and impulsivity would be positively associated with drive-to-eat behaviors, including hunger, food craving, and food cue responsiveness, consistent with evidence linking emotion regulation difficulties to craving and food consumption as well as impulsivity to sensitivity to rewarding food stimuli and cue-driven eating (Evers et al., 2018; Leehr et al., 2015; Schag, Schönleber et al., 2013a; Schag, Teufel et al., 2013b); (H3) drive-to-eat processes would be positively associated with higher levels of eating disorder severity and psychological distress (anxiety–depression, obsessive-compulsive symptoms, and perceived stress), in line with evidence linking appetitive motivational processes to binge eating and eating-related psychopathology (Boswell and Kober, 2016; Stojek and MacKillop, 2017; Udo and Grilo, 2019; Wonderlich et al., 2017); and (H4) emotion regulation and impulsivity would mediate the relationships between sex and age with both drive-to-eat behaviors and psychopathological outcomes, reflecting the proposed multilevel framework in which demographic characteristics may operate through relatively stable psychological characteristics to influence appetitive processes and clinical manifestations.

## 2 Methods

The present study analyzed baseline data from a clinical sample of patients with binge eating spectrum disorders recruited from a psychiatric service.

### 2.1 Participants

The total sample comprised 187 participants who met DSM-5 criteria for BSED for at least during the last 2 months. The disorder subtype was distributed as follow: *N* = 41 BN, *N* = 35 BED, *N* = 30 NES, and *N* = 81 OSFED. The OSFED group included participants who presented with binge-eating episodes and related symptoms but did not meet the full diagnostic criteria for BN, BED, or NES. Participants with restrictive eating disorder presentations, including atypical anorexia nervosa, were not included in this group. The patients were consecutively recruited from the psychiatry outpatient unit of the Citizen “Mental Health” Program, Ministry of Public Health of Ecuador. Age was into the range 20–45 years. Exclusion criteria included: (a) psychoactive substance dependence; (b) medical or neurological conditions causing severe cognitive impairment (e.g., Alzheimer’s disease); and (c) current use of psychotropic medication.

### 2.2 Measures

*Eating Disorder Severity*. Disordered eating behaviors and their psychological correlates were assessed using the Eating Disorder Inventory–3 (EDI-3; Garner and Van Strien, 2004), using the Spanish adapted version (Lizana-Calderón et al., 2022). The EDI-3 is a validated 91-item self-report questionnaire designed to assess core eating disorder symptoms and broader psychological features associated with eating disorders. Items are rated on a 6-point frequency scale ranging from 0 (“Never”) to 5 (“Always”). Higher scores indicate greater severity of the corresponding eating disorder symptoms and psychological characteristics.

*Food Craving*. Momentary food craving, defined as a temporary and context-dependent urge to eat, was measured using the Brief Form of the Food Craving Questionnaire–State (FCQ-S Brief; Meule, 2020), using the adapted Spanish version (Cepeda-Benito et al., 2000). This 10-item self-report instrument assesses cognitive, emotional, and physiological aspects of food craving. Items are rated on a 5-point response scale (1–5), with higher total scores indicating stronger food craving. Total scores range from 10 to 50.

*Hunger*. The transient experience of hunger, encompassing psychological and physiological components, was assessed using the Hunger subscale of the Three-Factor Eating Questionnaire (TFEQ; Stunkard and Messick, 1985), using the Spanish validation (Jáuregui-Lobera et al., 2014). This subscale assesses the intensity and frequency of hunger sensations and the tendency to eat in response to internal hunger cues. Items are rated on a 4-point response scale ranging from 1 (“False”) to 4 (“True”), with higher total scores indicating greater subjective hunger and sensitivity to internal hunger signals.

*Food Cues*. Responsiveness to food-related stimuli was assessed using the Power of Food Scale (PFS; Lowe et al., 2009), using the Spanish version (Serier et al., 2019). This 15-item self-report measure evaluates the psychological impact of food in the environment, independently of physiological hunger. Items are rated on a 5-point Likert-type scale, with higher scores indicating a stronger motivational and hedonic response to food cues. Total scores range from 15 to 75.

*Emotion Regulation*. Cognitive strategies for managing emotional responses were assessed using the Emotion Regulation Questionnaire (ERQ; Gross and John, 2003), with the Spanish adaptation (Cabello et al., 2013; Moreta-Herrera et al., 2022). This 10-item instrument uses a 7-point Likert scale ranging from 1 (“Strongly disagree”) to 7 (“Strongly agree”) and assesses two emotion regulation strategies: cognitive reappraisal and expressive suppression. In the present study, a total score was calculated to provide an overall index of emotion regulation.

*Stress*. Perceived stress was measured using the Perceived Stress Scale (PSS-10; Cohen et al., 1983), using the Spanish validation (Ruisoto et al., 2020). This 10-item self-report instrument assesses the degree to which individuals perceive their lives as unpredictable, uncontrollable, or overwhelming during the previous month. Items are rated on a 5-point frequency scale ranging from 0 (“Never”) to 4 (“Very often”). Higher total scores indicate greater perceived stress.

*Anxiety and Depression*. Anxiety and depressive symptoms were assessed using the Goldberg Anxiety and Depression Scale (GADS; Goldberg et al., 1988), using the Spanish version (Reivan-Ortiz et al., 2019). This 18-item self-report measure uses dichotomous (“Yes”/“No”) responses and comprises separate anxiety and depression subscales, as well as a total score reflecting the overall severity of these symptoms. Higher scores indicate greater symptom severity.

*Obsessive-Compulsive Symptoms*. Obsessive-compulsive symptom severity was assessed using the Maudsley Obsessive-Compulsive Inventory (MOCI; Hodgson and Rachman, 1977), using the Spanish version (Ávila-Espada, 1986). This 30-item self-report questionnaire assesses common obsessive thoughts and compulsive behaviors. Items are answered using dichotomous (“Yes”/“No”) responses, with higher total scores indicating greater obsessive-compulsive symptom severity.

*Impulsivity*. Impulsive tendencies were assessed using the Plutchik Impulsivity Scale (PIS; Plutchik and Van Praag, 1989), using the Spanish version (Rubio et al., 1998). This 15-item self-report scale assesses aspects of impulsivity including self-control, planning, anticipation of consequences, physiological responses, and spontaneous behaviors. Items are rated on a 4-point frequency scale ranging from 0 (“Never”) to 3 (“Always”), with higher total scores indicating greater impulsivity.

*Sociodemographic Variables*. Participants reported sociodemographic information, including age, sex, and marital status.

### 2.3 Ethical considerations and procedure

The study was carried out in accordance with the latest revision of the Declaration of Helsinki. Ethical approval was obtained from the Research Ethics Committee of the Catholic University of Cuenca and from the Clinical Research Ethics Committee of the Ecuadorian Ministry of Public Health (approval code: CEISH-UCACUE-2024-064). All participants provided written informed consent before being enrolled in the study.

The diagnosis of BSED was established by experienced physicians and psychiatrists specialized in the evaluation and treatment of eating disorders. Data were collected between February and September 2023. To minimize missing responses, clinicians supervised and assisted participants while they completed the different assessment instruments.

### 2.4 Statistical analysis

Statistical analysis was conducted with Stata19 for Windows (StataCorp, 2025). First, four groups defined by the participants’ sex and age were compared through analysis of variance (ANOVA), with post-hoc pairwise comparisons to identify differences in the psychologic and drive-to-eat measures. Given the moderate and adequate sample sizes across the four groups (N = 30–67), formal tests of normality (Kolmogorov–Smirnov or Shapiro–Wilk) and homogeneity of variances were not performed. This decision was supported by the robustness of the ANOVA to departures from normality: current studies based on Monte-Carlo simulations aimed to examine the performance of the F-test under a wide range of non-normal distributions and sample-size conditions, including both equal and unequal group sizes and different distributional shapes, have showed that the F-test maintained adequate Type I error control across all conditions examined, supporting the use of ANOVA when moderate deviations from normality are present (Blanca et al., 2017). The effect sizes for the contrasts were calculated through the standardized Cohen’s-*d* statistic, interpreting mild–moderate effect size for |*d*| > 0.50 and high-large effect for |*d*| > 0.80 (Cohen, 1988). In addition, to control for Type I error inflation due to multiple statistical testing, the Finner stepwise adjustment procedure was applied (Finner and Roters, 2002).

Path analyses were conducted within a structural equation modeling (SEM) framework, which enables the simultaneous estimation of multiple associations and the evaluation of both direct and indirect effects. This approach was used to investigate the contribution of sex and age to the relationships among impulsivity, emotion regulation, drive-to-eat variables (food cues, food craving, and hunger), and psychopathological outcomes (eating disorder severity, anxiety–depression, obsessive-compulsive symptoms, and perceived stress). Parameters were estimated using the maximum likelihood method, with all model parameters freely estimated to allow the model to reproduce the observed covariance matrix as closely as possible. Because the questionnaires included numerous subscales, total scores were used for each instrument to simplify the analytical structure.

Psychological symptom variables were specified as outcome variables, given their broad and state-like nature and their conceptual interpretation, from a clinical standpoint, as potential consequences of the remaining psychological and eating-related characteristics. In contrast, constructs such as impulsivity and emotion dysregulation, which are generally considered more stable traits, were specified as mediating variables, also influencing drive-to-eat constructs. An initial model was specified including all theoretically plausible direct and indirect paths among the study variables, together with covariances among exogenous variables, consistent with the exploratory objective of the study. Subsequently, non-significant paths were removed to obtain a more parsimonious and interpretable final model. Model adequacy was evaluated using commonly recommended fit indices (Barrett, 2007): the chi-square test (χ^2^), where non-significant values indicate good fit; the Standardized Root Mean Square Residual (SRMR), with values ≤ 0.10 considered acceptable; the Root Mean Square Error of Approximation (RMSEA), with values ≤ 0.08 indicating adequate fit; the Comparative Fit Index (CFI), with values ≥ 0.90 suggesting satisfactory fit; and the Tucker–Lewis Index (TLI), where values ≥ 0.90 are interpreted as reflecting adequate model fit.

## 3 Results

Table 1 displays the descriptive for all the variables analyzed in the study, among the completes sample and stratified by the diagnostic subtype. Overall, 63.6% of the participants were women (*n* = 119) and 36.4% were men (*n* = 68). Regarding marital status, most participants were married or in a couple relationship (64.7%, *n* = 121), followed by single participants (29.4%, *n* = 55), and a smaller proportion who were divorced or separated (5.9%, *n* = 11). The mean age of the sample was 31.82 years (*SD* = 5.76). Comparisons across diagnostic subtypes indicated that there were no statistically significant differences between groups in sex distribution, marital status, age, or any of the psychological variables assessed, suggesting that the diagnostic groups were comparable across the variables examined in this study.

**Table 1:** Descriptive for the variables analyzed in the study.

	Total *(N = 187)*		BN *(N = 41)*		BED *(N = 35)*		NES *(N = 30)*		OSFED *(N = 81)*		
	*n*	*%*	*n*	*%*	*n*	*%*	*n*	*%*	*n*	*%*	*p*
Sex											
Women	119	63.6%	26	63.4%	22	62.9%	21	70.0%	50	61.7%	0.882
Men	68	36.4%	15	36.6%	13	37.1%	9	30.0%	31	38.3%	
Marital status											
Single	55	29.4%	8	19.5%	16	45.7%	6	20.0%	25	30.9%	0.055
Married – couple	121	64.7%	33	80.5%	17	48.6%	21	70.0%	50	61.7%	
Divorced – separated	11	5.9%	0	0.0%	2	5.7%	3	10.0%	6	7.4%	

	*Mean*	*SD*	*Mean*	*SD*	*Mean*	*SD*	*Mean*	*SD*	*Mean*	*SD*	*p*
Age (years)	31.82	5.76	31.39	5.91	32.40	5.74	31.67	5.66	31.84	5.82	0.897
Emotion regulation	12.81	4.50	12.76	3.88	13.06	4.56	13.47	5.70	12.48	4.33	0.761
Impulsiveness	14.64	4.63	14.93	4.98	14.97	4.95	14.70	4.89	14.32	4.25	0.869
Hunger	19.09	6.83	19.07	7.19	19.03	6.55	20.03	7.31	18.77	6.67	0.861
Food craving	13.93	3.58	14.37	3.95	13.54	3.79	14.13	3.34	13.80	3.41	0.752
Food cues	16.02	4.24	15.71	3.90	15.89	4.63	16.33	4.20	16.12	4.31	0.927
Anxiety-Depression	14.97	5.40	15.32	5.42	15.37	5.84	15.60	6.09	14.38	4.96	0.634
ED severity	5.12	2.49	5.37	2.74	5.03	2.23	5.57	3.32	4.88	2.11	0.539
Obsessive-Compulsive	9.31	3.87	9.98	4.16	9.11	4.24	9.30	4.66	9.06	3.22	0.655
Stress	23.78	8.34	24.02	8.58	24.89	9.18	24.80	9.83	22.80	7.22	0.533

ED: eating disorder. BN: bulimia nervosa. BED: binge eating disorder. NES: night eating syndrome. OSFED: other specified feeding eating disorder.

Emotion (dys)regulation: ERQ total. Impulsivity: PIS total. Hunger: TFEQ total. Food craving: FCQ-S total. Food cues: PFS total. Anxiety-Depression: GADS total.

ED severity: EDI-3 total. Obsessive-Compulsive: MOCI total. Stress: PSS-10 total. SD: standard deviation. *Bold: significant comparison.

## 4 Differences between the groups defined by sex and age

Table 2 displays the results of the comparison across the four groups defined by sex and age (see also the radar-chart in Figure 2): women aged 20–30 years (*n* = 52), women aged 31–45 years (*n* = 67), men aged 20–30 years (*n* = 38), and men aged 31–45 years (*n* = 30). Significant differences emerged for hunger and food cue responsiveness. Hunger scores were significantly higher in women aged 20–30 years compared with men aged 31–45 years (*p* = 0.043), with a moderate effect size (|*d*| = 0.54). A similar pattern was observed when comparing women aged 31–45 years with men aged 31–45 years, with women reporting higher hunger levels (*p* = 0.016, |*d*| = 0.54). Food cue responsiveness also differed significantly between groups: women aged 31–45 years reported higher mean than women aged 20–30 years (*p* = 0.040, |*d*| = 0.37), and men aged 20–30 years showed significantly higher mean as compared to women aged 20–30 years (*p* = 0.003, |*d*| = 0.69) and women aged 31–45 years (*p* = 0.035, |*d*| = 0.56). No significant differences were observed between the remaining pairwise comparisons in emotion regulation, impulsiveness, anxiety–depression, eating disorder severity, obsessive-compulsive symptoms, or perceived stress.

**Table 2:** Association of the psychological measures with the groups defined by sex and age.

	Women		Women		Men		Men		Women 20–30		Women 20–30		Women 20–30		Women 31–45		Women 31–45		Men 20–30	
	20–30 yrs		31–45 yrs		20–30 yrs		31–45 yrs		*versus*		*versus*		*versus*		*versus*		*versus*		*versus*	
	*(N = 52)*		*(N = 67)*		*(N = 38)*		*(N = 30)*		Women 31–45		Men 20–30		Men 31–45		Men 20–30		Men 31–45		Men 31–45	
	*Mean*	*SD*	*Mean*	*SD*	*Mean*	*SD*	*Mean*	*SD*	*p*	*|d|*	*p*	*|d|*	*p*	*|d|*	*p*	*|d|*	*p*	*|d|*	*p*	*|d|*
Emotion regulation	13.17	4.46	13.09	4.63	12.26	4.08	12.23	4.90	0.920	0.02	0.347	0.21	0.366	0.20	0.369	0.19	0.390	0.18	0.978	0.01
Impulsiveness	13.60	5.10	15.18	4.36	14.42	4.43	15.50	4.46	0.065	0.33	0.403	0.17	0.073	0.40	0.419	0.17	0.752	0.07	0.339	0.24
Hunger	19.75	6.48	20.21	8.02	18.16	5.65	16.60	5.14	0.713	0.06	0.271	0.26	0.043*	**0.54** ^**†**^	0.136	0.30	0.016*	**0.54** ^†^	0.346	0.29
Food craving	13.19	3.64	14.45	3.97	14.16	3.02	13.77	3.13	0.059	0.33	0.207	0.29	0.484	0.17	0.690	0.08	0.387	0.19	0.655	0.13
Food cues	14.58	4.03	16.16	4.44	17.29	3.81	16.60	3.16	0.040*	0.37	0.003*	**0.69** ^†^	0.035*	**0.56** ^†^	0.184	0.27	0.634	0.11	0.498	0.20
Anxiety-Depression	15.65	5.83	15.22	5.39	14.13	4.98	14.27	5.21	0.668	0.08	0.189	0.28	0.265	0.25	0.322	0.21	0.422	0.18	0.919	0.03
ED severity	5.13	3.06	5.10	2.15	5.16	2.78	5.10	1.77	0.948	0.01	0.965	0.01	0.952	0.01	0.917	0.02	0.994	0.00	0.925	0.02
Obsessive-Comp.	9.44	4.22	10.03	4.13	8.53	3.28	8.47	3.09	0.410	0.14	0.266	0.24	0.270	0.26	0.056	0.40	0.066	0.43	0.949	0.02
Stress	23.73	9.35	25.09	8.93	22.58	6.86	22.47	6.60	0.379	0.15	0.518	0.14	0.509	0.16	0.140	0.32	0.154	0.33	0.956	0.02

Emotion (dys)regulation: ERQ total. Impulsivity: PIS total. Hunger: TFEQ total. Food craving: FCQ-S total. Food cues: PFS total. Anxiety-Depression: GADS total.

ED severity: EDI-3 total. Obsessive-Compulsive: MOCI total. Stress: PSS-10 total. SD: standard deviation.

*Bold: significant comparison. ^†^Bold: effect size within the mild–moderate to high-large range.

**Figure 2:**
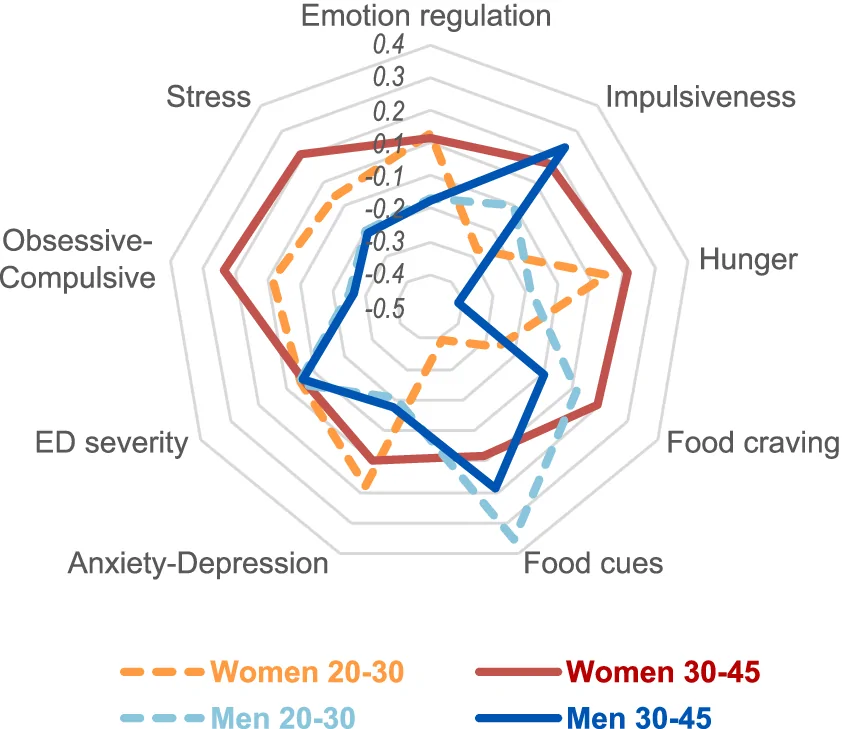
Radar chart with the standardized means across the groups defined by sex and age. Emotion (dys)regulation: ERQ total. Impulsivity: PIS total. Hunger: TFEQ total. Food craving: FCQ-S total. Food cues: PFS total. Anxiety-Depression: GADS total. ED severity: EDI-3 total. Obsessive-Compulsive: MOCI total. Stress: PSS-10 total.

### 4.1 Path analysis

Table 3 presents the heatmap of the correlation matrix for the variables included in the SEM. Overall, the pattern of associations indicated several significant correlations among the variables, which supports the inclusion of these features in the path analysis. Firstly, emotion (dys)regulation showed significant positive correlations with impulsiveness (*r* = 0.285, *p* < 0.001), food craving (*r* = 0.405, *p* < 0.001), anxiety–depression (*r* = 0.378, *p* < 0.001), eating disorder severity (*r* = 0.370, *p* < 0.001), obsessive-compulsive symptoms (*r* = 0.469, *p* < 0.001), and stress (*r* = 0.469, *p* < 0.001). Impulsiveness was positively associated with several variables, including hunger (*r* = 0.272, *p* < 0.001), food craving (*r* = 0.291, *p* < 0.001), food cue responsiveness (*r* = 0.534, *p* < 0.001), anxiety–depression (*r* = 0.359, *p* < 0.001), eating disorder severity (*r* = 0.557, *p* < 0.001), obsessive-compulsive symptoms (*r* = 0.600, *p* < 0.001), and stress (*r* = 0.600, *p* < 0.001). Hunger was positively correlated with food craving (*r* = 0.444, *p* < 0.001), anxiety–depression (*r* = 0.363, *p* < 0.001), eating disorder severity (*r* = 0.400, *p* < 0.001), obsessive-compulsive symptoms (*r* = 0.518, *p* < 0.001), and stress (*r* = 0.518, *p* < 0.001). Food craving showed significant positive correlations with food cue responsiveness (*r* = 0.192, *p* = 0.009), anxiety–depression (*r* = 0.215, *p* < 0.001), eating disorder severity (*r* = 0.281, *p* = 0.003), obsessive-compulsive symptoms (*r* = 0.268, *p* < 0.001), and stress (*r* = 0.268, *p* < 0.001). Food cue responsiveness was significantly associated with impulsiveness (*r* = 0.534, *p* < 0.001), food craving (*r* = 0.192, *p* = 0.009), anxiety–depression (*r* = 0.094, *p* = 0.048), obsessive-compulsive symptoms (*r* = 0.401, *p* < 0.001), and stress (*r* = 0.401, *p* < 0.001). Regarding the clinical outcomes, anxiety–depression, eating disorder severity, obsessive-compulsive symptoms, and stress were all significantly and positively intercorrelated (all *p* < 0.001).

**Table 3:** Heatmap of the correlation matrix for the variables analyzed in the SEM.

		1	2	3	4	5	6	7	8	9	10	11
1.	Gender		0.077	0.094	−0.043	0.179	−0.012	−0.172	0.109	−0.003	0.159	0.114
2.	Age	*0.297*		0.016	0.135	−0.016	0.125	0.036	−0.007	−0.006	0.091	0.068
3.	Emotion regulation	*0.202*	*0.830*		0.285	0.763	0.405	0.139	0.803	0.378	0.370	0.469
4.	Impulsiveness	*0.562*	*0.065*	*<0.001*		0.272	0.291	0.534	0.240	0.359	0.557	0.600
5.	Hunger	*0.014*	*0.826*	*<0.001*	*<0.001*		0.444	0.110	0.767	0.363	0.400	0.518
6.	Food craving	*0.875*	*0.088*	*<0.001*	*<0.001*	*<0.001*		0.192	0.391	0.215	0.281	0.268
7.	Food cues	*0.018*	*0.620*	*0.057*	*<0.001*	*0.134*	*0.009*		0.145	0.094	0.385	0.401
8.	Anxiety-Depression	*0.138*	*0.927*	*<0.001*	*0.001*	*<0.001*	*<0.001*	*0.048*		0.328	0.355	0.452
9.	ED severity	*0.969*	*0.940*	*<0.001*	*<0.001*	*<0.001*	*0.003*	*0.202*	*<0.001*		0.417	0.483
10.	Obsessive-Compulsive	*0.030*	*0.217*	*<0.001*	*<0.001*	*<0.001*	*<0.001*	*<0.001*	*<0.001*	*<0.001*		0.742
11.	Stress	*0.121*	*0.358*	*<0.001*	*<0.001*	*<0.001*	*<0.001*	*<0.001*	*<0.001*	*<0.001*	*<0.001*	

Upper diagonal part (normal font): correlation coefficients. Lower diagonal part (italics font): *p*-vlaues.

Emotion (dys)regulation: ERQ total. Impulsivity: PIS total. Hunger: TFEQ total. Food craving: FCQ-S total. Food cues: PFS total.

Anxiety-Depression: GADS total. ED severity: EDI-3 total. Obsessive-Compulsive: MOCI total. Stress: PSS-10 total.

Figure 3 shows the path diagram with the standardized coefficients obtained in the SEM (Table 4 contains the complete parameters). Adequate goodness of fit was achieved: χ^2^ = 25.53 (*p* = 0.699), RMSEA = 0.020 (95% confidence interval: 0.010 to 0.044), CFI = 0.999, TLI = 0.999 and SRMR = 0.033.

**Figure 3:**
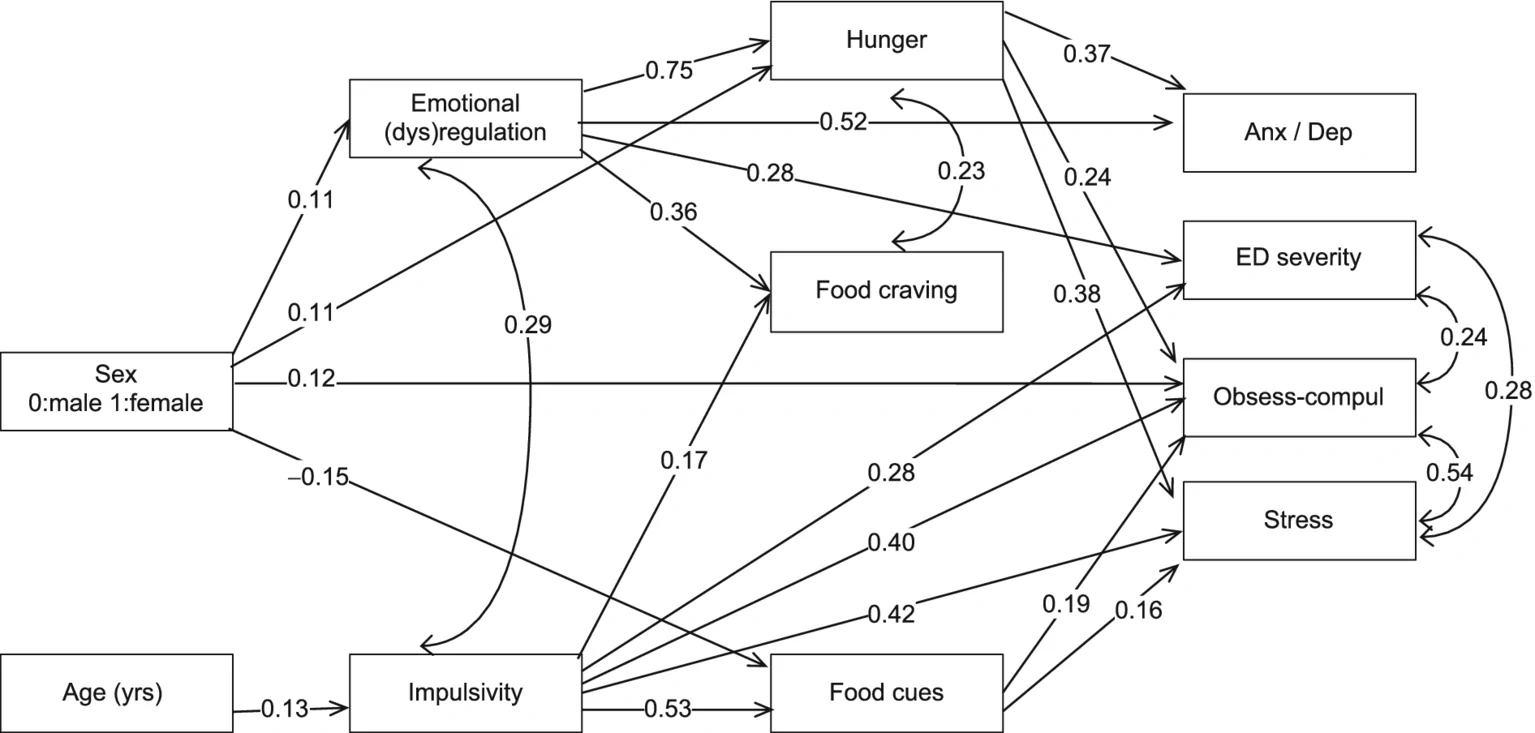
Path diagram with the standardized coefficients obtained in the SEM. Emotion (dys)regulation: ERQ total. Impulsivity: PIS total. Hunger: TFEQ total. Food craving: FCQ-S total. Food cues: PFS total. Anxiety-Depression: GADS total. ED severity: EDI-3 total. Obsessive-Compulsive: MOCI total. Stress: PSS-10 total.

**Table 4:** Standardized coefficients obtained in the SEM.

		Coeff.	SE	z-stat.	*p*	95%CI Coeff.	
Food cues	Impulsivity	0.5297	0.0523	10.13	<0.001	0.4272	0.6322
	Gender (female)	−0.1504	0.0605	−2.48	0.013	−0.2690	−0.0318
Hunger	Emotional (dys)regulation	0.7515	0.0316	23.80	<0.001	0.6896	0.8134
	Gender (female)	0.1145	0.0452	2.53	0.011	0.0260	0.2031
Emotional (dys)regulation	Gender (female)	0.1092	0.0689	1.58	0.113	−0.0259	0.2442
Obss-comp	Food Cues	0.1851	0.0656	2.82	0.005	0.0566	0.3135
	Hunger	0.2381	0.0590	4.04	<0.001	0.1225	0.3537
	Impulsivity	0.4033	0.0658	6.13	<0.001	0.2744	0.5322
	Gender (female)	0.1157	0.0495	2.34	0.019	0.0187	0.2127
Impulsivity	Age (yrs)	0.1331	0.0685	1.98	0.049	0.0011	0.2672
Stress	Food Cues	0.1621	0.0590	2.75	0.006	0.0465	0.2777
	Hunger	0.3781	0.0524	7.22	<0.001	0.2754	0.4808
	Impulsivity	0.4178	0.0608	6.87	<0.001	0.2986	0.5370
Anxiety-depression	Hunger	0.3696	0.0608	6.08	<0.001	0.2504	0.4889
	Emotional (dys)regulation	0.5208	0.0610	8.54	<0.001	0.4012	0.6403
Food craving	Emotional (dys)regulation	0.3582	0.0643	5.57	<0.001	0.2322	0.4841
	Impulsivity	0.1699	0.0666	2.55	0.011	0.0394	0.3004
ED severity	Emotional (dys)regulation	0.2833	0.0651	4.35	<0.001	0.1557	0.4109
	Impulsivity	0.2800	0.0655	4.28	<0.001	0.1516	0.4083
Covariances	Hunger; Food Craving	0.2273	0.0698	3.26	0.001	0.0905	0.3640
	Emotional (dys); Impulsivity	0.2924	0.0670	4.37	<0.001	0.1611	0.4236
	Obsess-Comp.; Stress	0.5409	0.0521	10.38	<0.001	0.4388	0.6430
	Obsess-Comp.; ED severity	0.2350	0.0701	3.35	0.001	0.0976	0.3725
	Stress.; ED severity	0.2765	0.0685	4.04	<0.001	0.1423	0.4107

Emotion (dys)regulation: ERQ total. Impulsivity: PIS total. Hunger: TFEQ total. Food craving: FCQ-S total. Food cues: PFS total.

Anxiety-Depression: GADS total. ED severity: EDI-3 total. Obsessive-Compulsive: MOCI total. Stress: PSS-10 total.

Some direct effects of sex and age were observed on psychological variables. Being women was associated with increased emotional regulation deficits, higher hunger and food cue levels, and greater obsessive-compulsive symptoms. Older age was also related to higher impulsivity scores.

Indirect effects of sex and age on drive-to-eat behaviors and psychopathology also emerged through the mediating role of emotional regulation and impulsivity. Specifically, being women was associated with greater emotional regulation impairment, which in turn contributed to higher hunger and food craving levels. In contrast, older age was associated with higher impulsivity, which subsequently contributed to increased food cue reactivity. Moreover, the pathways identified for women with greater emotional regulation difficulties were linked to higher anxiety–depression levels and greater eating disorder severity. Increased hunger levels also contributed to these pathways, further increasing anxiety–depression scores. Another pathway was observed for men with higher food cue levels, which were associated with greater obsessive-compulsive symptoms and perceived stress. Regarding age, a pathway was observed for older individuals with higher impulsivity, which was subsequently associated with greater eating disorder severity, obsessive-compulsive symptoms, and perceived stress. Food cues also contributed to this pathway, increasing obsessive-compulsive symptoms and perceived stress levels. Supplementary Table S1 presents the complete results for the direct, indirect, and total effects of the model.

## 5 Discussion

The present study investigated the contribution of sex and age to the clinical expression of BSED in young adults, focusing on the relationships among emotion regulation, impulsivity, drive-to-eat processes, and psychological distress. Overall, the findings suggest that although the general psychological profile of BSED appears relatively consistent across demographic groups, sex and age are associated with specific differences in appetite-related responses. Moreover, the results indicate that emotion regulation difficulties and impulsivity may represent key mechanisms through which demographic characteristics influence eating-related behaviors and psychological outcomes.

### 5.1 Sex and age differences in drive-to-eat behaviors

The comparison across groups defined by sex and age revealed significant differences only for hunger and responsiveness to food cues. Women reported higher levels of hunger than men, particularly when compared with men in the older age group. These findings are consistent with previous research suggesting that women may exhibit greater interoceptive sensibility or heightened awareness of bodily signals related to food intake (Becker et al., 2017; Garfinkel et al., 2015; Grabauskaitė et al., 2017; Nagata et al., 2023). In the context of BSED, heightened subjective hunger may reflect a complex interaction between physiological signals, emotional states, and cognitive processes related to eating behavior, including altered interoceptive processing, reward sensitivity, and emotion-driven regulation of intake (Hazzard et al., 2026; Kober and Boswell, 2018).

The observed sex differences in hunger may also be influenced by sociocultural and psychological factors, including greater body awareness and attentional focus on internal states among women (Cobbaert et al., 2026; Oswald et al., 2017). It is also possible that women are more likely to interpret or label interoceptive signals as hunger, particularly in contexts of emotional distress or dietary restraint. This is especially relevant in BSED, where difficulties in distinguishing between physiological hunger and emotionally driven urges to eat have been widely documented (Glenk et al., 2018).

Interestingly, these differences were more pronounced when women were compared to men in the older age group, suggesting a potential interaction between sex and developmental stage. One possible explanation is that age-related improvements in self-regulation may differentially benefit men, leading to a reduction in perceived hunger or reactivity to internal cues over time. In contrast, women may remain more sensitive to these internal states or may experience persistent vulnerability due to factors such as chronic dieting, body dissatisfaction, or stress-related eating patterns (Rohde et al., 2015). From a neurobehavioral perspective, heightened hunger in women could also reflect greater integration between homeostatic and hedonic signals, whereby internal physiological states more readily trigger motivational processes related to food intake (Kober and Boswell, 2018). This interpretation aligns with models of binge eating that emphasize increased salience of food-related cues and altered processing of reward signals (Bodell et al., 2018; Chao et al., 2024).

In contrast, food cue responsiveness appeared to be particularly elevated among younger men. This result suggests that environmental stimuli associated with food may exert a stronger motivational influence on eating behavior in this subgroup. Previous research has indicated that cue-driven eating may play an important role in overeating and binge episodes, particularly in individuals with higher reward sensitivity (Boswell and Kober, 2016; Hayashi et al., 2023). One possible explanation for the heightened cue responsiveness observed in younger men relates to developmental differences in reward processing. During late adolescence and early adulthood, heightened sensitivity to reward and novelty, combined with still-maturing regulatory control systems, may increase susceptibility to salient environmental cues. In this context, palatable food cues may acquire enhanced motivational value, triggering approach-oriented behaviors even in the absence of physiological hunger (De Cock et al., 2016; Harden et al., 2018). Additionally, sex-related differences in attentional biases and motivational salience attributed to food stimuli may further contribute to this pattern (Buczek et al., 2020). Younger men may be more prone to externally driven eating, relying more heavily on immediate environmental signals rather than internal states to guide food intake. This tendency could be exacerbated in highly obesogenic environments, where energy-dense foods are widely available and frequently promoted (Shteyn and Petrovich, 2025).

Importantly, these findings complement the observed pattern in women, suggesting a potential divergence in the dominant drivers of eating behavior: whereas women may be more influenced by internal states such as hunger and emotional processes, younger men may be more reactive to external food-related cues. Such differentiation aligns with dual-process models of eating behavior, in which behavior is shaped by the dynamic interplay between internally driven (homeostatic and emotional) and externally driven (cue-reactive and reward-based) mechanisms (Gao et al., 2026; Morton et al., 2014).

Interestingly, no significant group differences were observed in emotion regulation, impulsivity, eating disorder severity, or broader psychopathological symptoms. One possible interpretation is that processes such as emotion dysregulation, impulsivity, and general psychopathology may represent transdiagnostic features of BSED that are already consolidated by early adulthood, thereby reducing the likelihood of detecting differences across demographic strata. Alternatively, it is also possible that the measures employed capture relatively stable trait-like characteristics, which may be less sensitive to developmental or sex-related variations compared to more state-dependent processes such as hunger or cue reactivity. Finally, these findings reinforce the idea that BSED share common psychological mechanisms despite variability in demographic characteristics (Carr et al., 2021; Meier et al., 2025).

### 5.2 Psychological mechanisms underlying binge eating behaviors

The correlation patterns and path analysis results provide further insight into the relationships among psychological traits, drive-to-eat processes, and psychopathological outcomes. As expected, emotion regulation difficulties were positively associated with multiple clinical variables, including impulsivity, food craving, eating disorder severity, and psychological distress. These associations support previous evidence indicating that impaired emotion regulation represents a central vulnerability factor in binge eating and related psychopathology (Arexis et al., 2023; Borm et al., 2025). Similarly, impulsivity showed strong associations with several drive-to-eat measures and clinical outcomes. In particular, impulsivity was strongly related to food cue responsiveness, as well as to eating disorder severity and obsessive-compulsive symptoms. These findings align with theoretical models suggesting that impulsivity may increase susceptibility to reward-related stimuli, thereby facilitating cue-driven eating behaviors and difficulties in controlling food intake (Hege et al., 2015; Michael and Juarascio, 2021; Schag et al., 2013a; Schag et al., 2013b).

Importantly, the SEM provided evidence that emotion regulation and impulsivity operate as mediating mechanisms linking demographic characteristics to appetitive responses and psychological outcomes. Being female was associated with greater emotion regulation difficulties, which in turn contributed to higher levels of hunger and food craving. These processes were further linked to increased anxiety–depression and eating disorder severity, suggesting that emotion-related pathways may represent a particularly relevant mechanism underlying binge eating behaviors among women. This pattern is consistent with models of binge eating that emphasize the role of affect regulation in the initiation and maintenance of maladaptive eating behaviors, whereby negative emotional states and difficulties in downregulating affective arousal increase reliance on food intake as a coping strategy. In this context, hunger and craving may reflect not only physiological or hedonic states, but also a dysregulated interface between emotional distress and reward-related processes (Devonport et al., 2019; Evers et al., 2018).

In contrast, age-related effects appeared to operate primarily through impulsivity. Although the bivariate association between age and impulsivity was small and did not reach statistical significance (*r* = 0.135, *p* = 0.065), the path analysis identified a positive age-to-impulsivity pathway within the multivariable model. Within this pathway, higher impulsivity was associated with greater responsiveness to food cues and subsequently with increased psychopathological symptoms, including obsessive-compulsive traits and perceived stress. Notably, food cue responsiveness emerged as a pivotal downstream mechanism in this pathway, reinforcing the role of environmental food stimuli in amplifying both motivational salience and psychological distress. This finding aligns with incentive-sensitization accounts of overeating, which posit that repeated exposure to palatable food cues can strengthen cue–reward associations, thereby increasing compulsive-like eating tendencies even in the absence of homeostatic need (Berridge and Robinson, 2016; DeHart et al., 2020; Thanarajah et al., 2019).

The positive age-to-impulsivity pathway should nevertheless be interpreted cautiously, as it differs from the developmental pattern typically reported in which impulsivity tends to be higher at younger ages and declines across adulthood (Ray and Jones, 2023). Several factors may account for this apparently discrepant finding. First, the present sample comprised young adults with BSED rather than a population-based sample, and therefore the observed pathway may reflect characteristics specific to the clinical population rather than a general developmental association between age and impulsivity. Second, the relatively restricted age range (20–45 years) may capture individual differences in the clinical expression and persistence of impulsive traits rather than the broader developmental changes in self-regulation typically observed across adolescence and adulthood. Importantly, the non-significant bivariate association between age and impulsivity indicates that the positive pathway identified in the path analysis should not be interpreted as evidence of a general age-related increase in impulsivity. Rather, it may reflect the role of impulsivity within the multivariable network linking age to appetitive and psychopathological outcomes.

An additional pathway was observed among men, whereby elevated food cue responsiveness was associated with higher obsessive-compulsive symptoms and perceived stress. This finding further supports the idea that cue-driven eating processes may play a particularly relevant role in the expression of binge eating-related behaviors in men (Boswell and Kober, 2016). Importantly, this association suggests that food cue reactivity in men may extend beyond eating-specific outcomes and reflect a broader sensitivity to external stimuli that is linked to heightened psychological distress. One possible interpretation is that increased responsiveness to food-related cues may index a more general difficulty in modulating stimulus-driven behavioral tendencies, which in turn could contribute to both compulsive-like cognitive patterns and elevated stress perception (Robinson et al., 2016; Robinson and Berridge, 2025). Within this framework, food cues may act as salient environmental triggers that not only promote eating behavior, but also interact with broader dimensions of cognitive control and affective regulation.

These findings are in line with conceptualizations of binge-spectrum behaviors that emphasize the role of cue-reactivity as a transdiagnostic process, potentially shared across compulsive and stress-related psychopathology. In men, this may manifest more strongly through externally oriented processes, whereby environmental salience drives both behavioral and emotional dysregulation.

### 5.3 Clinical implications and future directions

The present findings have several important implications for the assessment and treatment of BSED. First, the observed differences in drive-to-eat processes (particularly hunger and responsiveness to food-related cues) suggest that motivational eating-related states are not uniform across individuals but may vary as a function of sex and age. This highlights the clinical relevance of systematically assessing both internally driven (e.g., hunger, affective states) and externally driven (e.g., cue-reactivity) eating processes, as these may differentially contribute to symptom expression and maintenance across demographic groups.

Second, the mediating roles of emotion regulation and impulsivity underscore these constructs as key transdiagnostic targets for intervention. In particular, difficulties in modulating negative affect and inhibitory control appear to represent proximal mechanisms linking appetitive processes with broader psychopathological outcomes and eating disorder severity. These findings support the integration of emotion-focused and executive control–based strategies within treatment approaches. For example, interventions that enhance emotional awareness, distress tolerance, and cognitive reappraisal skills, alongside training in inhibitory control and response delay, may be especially effective in reducing vulnerability to craving-driven and cue-elicited eating.

Third, the results suggest potential clinical utility in addressing cue-reactivity directly. Approaches aimed at modifying learned associations between environmental food cues and reward valuation (such as cue exposure paradigms, attentional bias modification, or strategies targeting reward revaluation) may be particularly relevant for individuals exhibiting heightened external responsiveness. In this context, strengthening top-down regulatory control may also buffer against the impact of obesogenic environments characterized by persistent exposure to palatable food cues.

More broadly, the findings support an integrated clinical framework in which binge-spectrum eating is conceptualized as the product of dynamic interactions between stable trait-like vulnerabilities (e.g., impulsivity, emotion dysregulation) and more proximal motivational states (e.g., craving, hunger, cue-induced desire). Such a framework may facilitate more precise case formulation and the development of individualized treatment strategies across the binge eating spectrum.

### 5.4 Strengths and limitations

This study presents several strengths that enhance the robustness and interpretability of its findings. First, the inclusion of a relatively large and clinically diverse sample of individuals across the BSED (including BN, BED, NES, and OSFED with binge features) allowed for a transdiagnostic approach that captures the heterogeneity of binge-related pathology within a unified analytical framework. Second, the study simultaneously examined multiple theoretically relevant psychological domains, including emotion regulation, impulsivity, eating disorder severity, and drive-to-eat processes (food cues, craving, and hunger), as well as broader psychopathological indicators such as anxiety-depression, obsessive-compulsive symptoms, and perceived stress. This comprehensive assessment enabled a multidimensional characterization of BSED and their associated mechanisms. Third, the use of path analysis provided a more nuanced understanding of the direct and indirect relationships among sex, age, psychological variables, and eating-related behaviors. This analytical approach allowed for the testing of theoretically grounded mediation pathways, offering insights into potential mechanisms underlying demographic differences in BSED expression. Fourth, the inclusion of both sex and age as key demographic variables represents an additional strength, as it enabled the examination of developmental and biological influences within the same analytical model, which is often lacking in binge eating research. Finally, by focusing on young adults, a developmental period characterized by heightened vulnerability to reward sensitivity and ongoing maturation of cognitive control systems, the study targets a clinically and developmentally relevant window for the emergence and consolidation of binge-spectrum eating behaviors. This enhances the translational relevance of the findings for early identification and intervention strategies.

However, some limitations should also be acknowledged. First, the cross-sectional design precludes conclusions about causal relationships between variables. Longitudinal studies would be necessary to determine the temporal direction of the observed associations. Second, the sample consisted exclusively of young adults within a restricted age range, which limits the generalizability of the findings to adolescents, middle-aged, or older populations. Given evidence that both reward sensitivity and cognitive control undergo developmental changes across the lifespan, the observed patterns may not extend to other age groups. Third, although the study included several relevant psychological domains, other potentially important variables were not assessed. In particular, factors such as dietary restraint, reward learning history, neurobiological markers of reward sensitivity, and environmental influences (e.g., food availability or sociocultural pressures) may further contribute to binge-spectrum eating pathology and could refine the proposed pathways. Fourth, the distribution of participants across the sex-by-age groups was not fully balanced, with group sizes ranging from 30 to 67 participants (the subgroup of men aged 31–45 years was the smallest, *N* = 30). Although these sample sizes allowed the planned comparisons to be conducted, the unequal group sizes may have reduced the precision of some estimates, particularly for the smallest subgroup. Therefore, findings concerning sex- and age-related differences should be interpreted cautiously and replicated in larger samples with more balanced representation across sex and age groups.

## 6 Conclusion

The present study highlights the role of sex and age in shaping specific aspects of the clinical presentation of BSED. While most psychological variables were comparable across demographic groups, differences emerged in key drive-to-eat processes, particularly hunger and responsiveness to food-related cues. These findings suggest that demographic factors may exert their influence on eating pathology primarily through specific appetitive pathways rather than broad psychopathological profiles.

The results further indicate that emotion regulation difficulties and impulsivity may represent relevant mechanisms linking demographic characteristics to appetitive responses and associated psychological distress. This pattern supports the relevance of integrating both affective and inhibitory control processes when conceptualizing vulnerability to BSED. Overall, these findings may contribute to more nuanced clinical assessment and the development of individualized, mechanism-informed interventions.

Future research should employ longitudinal designs to clarify the temporal and potentially bidirectional relationships among emotion regulation, impulsivity, and appetitive processes. Incorporating neurobiological and computational approaches to reward processing may further refine the understanding of sex- and age-related differences in cue responsiveness. In addition, examining how these mechanisms change in response to psychological interventions could advance knowledge of treatment processes and support the development of targeted, mechanism-based therapies for BSED.

## Data Availability

The raw data supporting the conclusions of this article will be made available by the authors, without undue reservation.
