# Supplementary material for: Sex and age effects on binge eating spectrum disorders in young adults: a path analysis
Source: Front Psychol. 2026 Sep 22;17:1870692. doi: 10.3389/fpsyg.2026.1870692 (PMC13638542; doi:10.3389/fpsyg.2026.1870692)
Supplement: Supplementary file 1 [file Supplementary_file_1.docx]

***Table S1 (supplementary).*** Complete results obtained in the SEM: direct, indirect and total effects

| Direct effects |  | *Coeff.* | *SE* | *z-stat.* | *p* | *Std.coeff.* |
| --- | --- | --- | --- | --- | --- | --- |
| Food Cues | Impulsivity | 0.4836 | 0.0558 | 8.66 | <0.001 | 0.5297 |
|  | Gender (female) | -1.3166 | 0.5356 | -2.46 | 0.014 | -0.1504 |
| Hunger | Emotional (dys)regulation | 1.1403 | 0.0709 | 16.09 | <0.001 | 0.7515 |
|  | Gender (female) | 1.6253 | 0.6455 | 2.52 | 0.012 | 0.1145 |
| Emotional (dys)regulation | Gender (female) | 1.0212 | 0.6513 | 1.57 | 0.117 | 0.1092 |
| Obss-Comp | Food Cues | 0.1681 | 0.0601 | 2.80 | 0.005 | 0.1851 |
|  | Hunger | 0.1335 | 0.0333 | 4.01 | <0.001 | 0.2381 |
|  | Impulsivity | 0.3345 | 0.0570 | 5.87 | <0.001 | 0.4033 |
|  | Gender (female) | 0.9201 | 0.3955 | 2.33 | 0.020 | 0.1157 |
| Impulsivity | Age (yrs) | 0.1068 | 0.0557 | 1.98 | 0.049 | 0.1331 |
| Stress | Food Cues | 0.3162 | 0.1157 | 2.73 | 0.006 | 0.1621 |
|  | Hunger | 0.4550 | 0.0643 | 7.07 | <0.001 | 0.3781 |
|  | Impulsivity | 0.7439 | 0.1119 | 6.65 | <0.001 | 0.4178 |
| Anxiety-Depression | Hunger | 0.2922 | 0.0490 | 5.96 | <0.001 | 0.3696 |
|  | Emotional (dys)regulation | 0.6246 | 0.0742 | 8.41 | <0.001 | 0.5208 |
| Food Craving | Emotional (dys)regulation | 0.2835 | 0.0543 | 5.22 | <0.001 | 0.3582 |
|  | Impulsivity | 0.1312 | 0.0518 | 2.53 | 0.011 | 0.1699 |
| ED severity | Emotional (dys)regulation | 0.1559 | 0.0369 | 4.23 | <0.001 | 0.2833 |
|  | Impulsivity | 0.1502 | 0.0364 | 4.12 | <0.001 | 0.2800 |
| Indirect effects |  | *Coeff.* | *SE* | *z-stat.* | *p* | *Std.coeff.* |
| Food Cues | Age (yrs) | 0.0517 | 0.0276 | 1.87 | 0.061 | 0.0705 |
| Hunger | Gender (female) | 1.1644 | 0.7462 | 1.56 | 0.119 | 0.0820 |
| Obss-Comp | Emotional (dys)regulation | 0.1522 | 0.0391 | 3.89 | <0.001 | 0.1789 |
|  | Impulsivity | 0.0813 | 0.0305 | 2.66 | 0.008 | 0.0980 |
|  | Gender (female) | 0.1509 | 0.2003 | 0.75 | 0.451 | 0.0190 |
|  | Age (yrs) | 0.0444 | 0.0237 | 1.87 | 0.061 | 0.0667 |
| Stress | Emotional (dys)regulation | 0.5188 | 0.0801 | 6.47 | <0.001 | 0.2842 |
|  | Impulsivity | 0.1529 | 0.0587 | 2.61 | 0.009 | 0.0859 |
|  | Gender (female) | 0.8529 | 0.5311 | 1.61 | 0.108 | 0.0499 |
|  | Age (yrs) | 0.0958 | 0.0510 | 1.88 | 0.060 | 0.0670 |
| Anxiety-Depression | Emotional (dys)regulation | 0.3332 | 0.0596 | 5.59 | <0.001 | 0.2778 |
|  | Gender (female) | 1.4529 | 0.6580 | 2.21 | 0.027 | 0.1295 |
| Food Craving | Gender (female) | 0.2895 | 0.1928 | 1.50 | 0.133 | 0.0391 |
|  | Age (yrs) | 0.0140 | 0.0092 | 1.53 | 0.126 | 0.0226 |
| ED severity | Gender (female) | 0.1592 | 0.1083 | 1.47 | 0.142 | 0.0309 |
|  | Age (yrs) | 0.0160 | 0.0092 | 1.74 | 0.082 | 0.0373 |
| Total effects |  | *Coeff.* | *SE* | *z-stat.* | *p* | *Std.coeff.* |
| Food Cues | Impulsivity | 0.4836 | 0.0558 | 8.66 | <0.001 | 0.5297 |
|  | Gender (female) | -1.3166 | 0.5356 | -2.46 | 0.014 | -0.1504 |
|  | Age (yrs) | 0.0517 | 0.0276 | 1.87 | 0.061 | 0.0705 |
| Hunger | Emotional (dys)regulation | 1.1403 | 0.0709 | 16.09 | <0.001 | 0.7515 |
|  | Gender (female) | 2.7897 | 0.9823 | 2.84 | 0.005 | 0.1966 |
| Emotional (dys)regulation | Gender (female) | 1.0212 | 0.6513 | 1.57 | 0.117 | 0.1092 |
| Obss-Comp | Food Cues | 0.1681 | 0.0601 | 2.80 | 0.005 | 0.1851 |
|  | Hunger | 0.1335 | 0.0333 | 4.01 | <0.001 | 0.2381 |
|  | Emotional (dys)regulation | 0.1522 | 0.0391 | 3.89 | <0.001 | 0.1789 |
|  | Impulsivity | 0.4158 | 0.0498 | 8.35 | <0.001 | 0.5013 |
|  | Gender (female) | 1.0710 | 0.4173 | 2.57 | 0.010 | 0.1346 |
|  | Age (yrs) | 0.0444 | 0.0237 | 1.87 | 0.061 | 0.0667 |
| Impulsivity | Age (yrs) | 0.1068 | 0.0557 | 1.92 | 0.055 | 0.1331 |
| Stress | Food Cues | 0.3162 | 0.1157 | 2.73 | 0.006 | 0.1621 |
|  | Hunger | 0.4550 | 0.0643 | 7.07 | <0.001 | 0.3781 |
|  | Emotional (dys)regulation | 0.5188 | 0.0801 | 6.47 | <0.001 | 0.2842 |
|  | Impulsivity | 0.8968 | 0.0977 | 9.18 | <0.001 | 0.5037 |
|  | Gender (female) | 0.8529 | 0.5311 | 1.61 | 0.108 | 0.0499 |
|  | Age (yrs) | 0.0958 | 0.0510 | 1.88 | 0.060 | 0.0670 |
| Anxiety-Depression | Hunger | 0.2922 | 0.0490 | 5.96 | <0.001 | 0.3696 |
|  | Emotional (dys)regulation | 0.9578 | 0.0523 | 18.33 | <0.001 | 0.7986 |
|  | Gender (female) | 1.4529 | 0.6580 | 2.21 | 0.027 | 0.1295 |
| Food Craving | Emotional (dys)regulation | 0.2835 | 0.0543 | 5.22 | <0.001 | 0.3582 |
|  | Impulsivity | 0.1312 | 0.0518 | 2.53 | 0.011 | 0.1699 |
|  | Gender (female) | 0.2895 | 0.1928 | 1.50 | 0.133 | 0.0391 |
|  | Age (yrs) | 0.0140 | 0.0092 | 1.53 | 0.126 | 0.0226 |
| ED severity | Emotional (dys)regulation | 0.1559 | 0.0369 | 4.23 | <0.001 | 0.2833 |
|  | Impulsivity | 0.1502 | 0.0364 | 4.12 | <0.001 | 0.2800 |
|  | Gender (female) | 0.1592 | 0.1083 | 1.47 | 0.142 | 0.0309 |
|  | Age (yrs) | 0.0160 | 0.0092 | 1.74 | 0.082 | 0.0373 |

*Note.* Emotion (dys)regulation: ERQ total. Impulsivity: PIS total. Hunger: TFEQ total. Food craving: FCQ-S total. Food cues: PFS total.

Anxiety-Depression: GADS total. ED severity: EDI-3 total. Obsessive-Compulsive: MOCI total. Stress: PSS-10 total.
